# Frequency conversion of structured light

**DOI:** 10.1038/srep21390

**Published:** 2016-02-15

**Authors:** Fabian Steinlechner, Nathaniel Hermosa, Valerio Pruneri, Juan P. Torres

**Affiliations:** 1 ICFO—Institut de Ciencies Fotoniques, The Barcelona Institute of Science and Technology, 08860 Castelldefels, Spain; 2 IQOQI—Institute for Quantum Optics and Quantum Information, Austrian Academy of Sciences, Boltzmanngasse 3, 1090 Wien, Austria; 3 National Institute of Physics, University of the Philippines Diliman, Quezon City, 1101 Philippines; 4 ICREA—Institució Catalana de Recerca i Estudis Avançats, 08010 Barcelona, Spain; 5 Department of Signal Theory and Communications, Polytechnic University of Catalonia, Jordi Girona 1-3, 08034 Barcelona, Spain

## Abstract

Coherent frequency conversion of structured light, i.e. the ability to manipulate the carrier frequency of a wave front without distorting its spatial phase and intensity profile, provides the opportunity for numerous novel applications in photonic technology and fundamental science. In particular, frequency conversion of spatial modes carrying orbital angular momentum can be exploited in sub-wavelength resolution nano-optics and coherent imaging at a wavelength different from that used to illuminate an object. Moreover, coherent frequency conversion will be crucial for interfacing information stored in the high-dimensional spatial structure of single and entangled photons with various constituents of quantum networks. In this work, we demonstrate frequency conversion of structured light from the near infrared (803 nm) to the visible (527 nm). The conversion scheme is based on sum-frequency generation in a periodically poled lithium niobate crystal pumped with a 1540-nm Gaussian beam. We observe frequency-converted fields that exhibit a high degree of similarity with the input field and verify the coherence of the frequency-conversion process via mode projection measurements with a phase mask and a single-mode fiber. Our results demonstrate the suitability of exploiting the technique for applications in quantum information processing and coherent imaging.

The efficient generation, manipulation, and detection of structured light are of great importance for numerous fields of research and technology, such as optical communications, quantum optics spectroscopy, and microscopy. In particular, beams with orbital angular momentum (OAM)^1^,^2^,^3^ have been used to enhance the channel capacity in optical free-space communications^4^, as optical tweezers^5^, in high-resolution nanoscopy^6^ and measurements with increased angular resolution^7^. Furthermore, quantum information carried in the rich high-dimensional spatial structure of single and entangled photons^8^,^9^,^10^ can be exploited in applications such as multi-dimensional quantum teleportation^11^.

The operational wavelength in such experiments is usually determined by the availability of light sources, the transmission wavelength of communication links, the absorption or reflection of the material probed, and detector sensitivity. Optical frequency conversion offers an elegant and practical way to overcome trade-offs associated with the use of a single operational wavelength and conflicting wavelength-dependent efficiencies of experimental components. As an example, frequency up-conversion^12^,^13^,^14^ from the infrared to the visible offers a convenient technique for extending the working range of efficient off-the-shelf single-photon detectors and low-noise CCDs. Coherent frequency conversion of high-dimensional quantum information carriers will also be an indispensable part of a quantum repeater infrastructure as it allows interfacing between the telecom bands or free-space transmission windows, and atomic quantum memories^15^,^16^,^17^,^18^,^19^,^20^,^21^.

An ideal frequency converter transfers light to a target wavelength without distorting the mode structure of the field, this being a particular polarization mode^22^,^23^, temporal mode^24^, or spatial mode with its characteristic intensity and phase profile. To this end, parametric processes in nonlinear materials^25^ offer several well-established methods for converting the frequency of light, such as sum-frequency generation (SFG), second-harmonic generation (SHG), difference frequency generation (DFG), and four-wave mixing (FWM). The frequency conversion^26^,^27^,^28^,^29^,^30^,^31^,^32^,^33^ and amplification^34^,^35^,^36^,^37^,^38^,^39^,^40^ of electromagnetic fields carrying spatial intensity information has been a key accomplishment in the field of nonlinear optics. Additionally, frequency conversion of spiral phase modes with a well-defined OAM value *l* has been successfully demonstrated for SHG^41^,^42^,^43^, SFG^44^,^45^, and four-wave mixing in atomic vapor^46^. A crucial requirement for the exploitation of frequency conversion in applications of spatially encoded quantum information is that the conversion process be coherent, i.e. it must be possible to convert superpositions of modes with different OAM. This issue can be addressed by frequency-converting Hermite-Gauss (HG) beams, which can be represented as a superposition of Laguerre-Gauss (LG^*l*^) modes with different OAM content *l*^47^, and employing a coherent detection scheme. Frequency conversion of beams in a superposition of different *l* has very recently been demonstrated by other groups for SHG^48^ and SFG^49^.

Here, we report on the frequency conversion of HG spatial modes, i.e. superpositions of modes with different OAM content, from the near infrared (803 nm) to the visible spectral range (527 nm). The motivation is two-fold: 1) this demonstrates the suitability of frequency-converting spatially encoded states for applications in quantum information processing; and, 2) it shows how spatial information stored in the phase of a structured light beam can be converted to other wavelengths, which may extend phase-sensitive imaging and spectroscopy into new wavelength regions.

The optical frequency converter presented here is based on SFG with a strong Gaussian pump beam. In order to efficiently frequency up-convert the phase information stored in the wave front with minimum aberrations, we employ two key technological developments. First, the use of a periodically poled nonlinear crystal, which allows the interaction to be tailored to a non-critical phase-matching configuration without spatial walk-off, while at the same time using the largest nonlinear coefficient of the material. Second, the availability of powerful off-the-shelf fibered pump sources near 1550 nm based on erbium-doped fiber amplifiers. In the succeeding part of the article, we show that the 527-nm sum-frequency field exhibits the expected characteristic far-field intensity profile of the 803-nm input beams, and verify the preservation of coherence between different OAM values using a mode projection technique with a phase mask and a single-mode fiber.

## Theoretical Background

In order to get an intuitive understanding of SFG with structured light, let us briefly discuss the expected spatial distribution of the output field using standard quantum optics formalism^50^. We assume that the pump is a monochromatic coherent field with a frequency 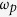, and that the signal field is a single-photon, or weak coherent state with a frequency 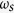. Our objective is to transfer the spatial structure of the signal field to the sum frequency mode, which is initially in its vacuum state. The signal field is described by its mode function in transverse momentum space 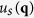. We assume that the pump is Gaussian beam 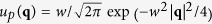 with its beam waist 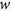 located at the center of the nonlinear crystal. We consider only the prototypical experimental scenario in which fields propagate along a principal crystallographic axis of a periodically poled nonlinear crystal of length *L* and nonlinear coefficient 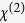. Due to energy and momentum conservation, the conditions 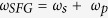, and 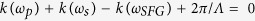 must hold, where 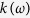 denotes the respective longitudinal wave vector for 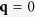, and 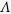 is the poling period of the nonlinear crystal. Under these conditions the spatial state of the sum-frequency field can be expressed as:


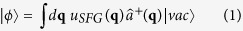
where 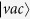 denotes the initial vacuum state of the sum-frequency mode, and 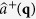 is the creation operator for a photon with a transverse momentum component ***q***. The spatial mode of the sum-frequency wave reads:


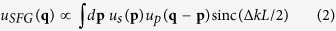
with a longitudinal phase-mismatch function 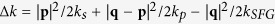. Due to symmetry, the total OAM is conserved for collinear SFG 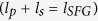. Also, since the Gaussian pump beam carries no OAM 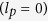, the OAM of the signal beam is transferred to the SFG field 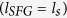. However, the spatial mode generated in the SFG process may be distorted with respect to the input mode as a result of spatial filtering due to: a) the limited spatial extent of the pump mode and b) the spatial bandwidth of the phase-matching function^51^. However, if the length of the nonlinear crystal is short compared to the diffraction length of the pump and signal beams, the spatial mode of the SFG yields a perfect frequency-converted replica of the signal field 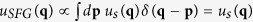. A detailed analysis of the effects of spatial filtering is beyond the scope of this article, and we restrict our considerations to the conservation of the phase structure of superposition of modes with different values of OAM. For a detailed discussion of the spatial transfer function in SFG and SHG, see Refs 36, 52, 53 and 54.

## Experimental Results

**Figure 1:**
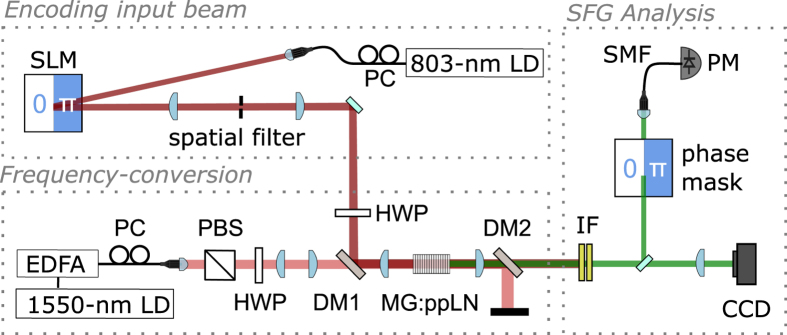
Experimental Setup. ***Encoding** the input beam*: The 803-nm input mode (dark red) is prepared by modulating the spatial phase of photons from a fiber-coupled laser diode (LD) via a spatial light modulator (SLM). *Frequency conversion*: A nonlinear crystal (Mg:ppLN) is pumped with a Gaussian beam emitted by from a fiber-coupled 1540-nm LD (light red). The pump power is controlled using a fiber polarization controller (PC) and a polarizing beam splitter. The polarization states of the pump and input modes are set using half-wave plates (HWP). The 1540-nm and the 803-nm light are combined via a dichroic mirror (DM1) and focused to the center of the nonlinear crystal via an achromatic lens. A second dichroic mirror (DM2) blocks the pump light, and transmits the 803-nm beam and the 527-nm sum-frequency mode (green). *SFG analysis*: The 803-nm light is blocked by an interference filter (IF) and the intensity profile of the sum-frequency field is analyzed using a CCD. The phase-structure of the SFG mode is analyzed using a phase mask and a single-mode fiber (SMF). The power coupled into the single-mode fiber is monitored using a power meter (PM).

The experimental setup depicted in Fig. 1 consists of three stages. First, the input laser (803 nm) is manipulated in order to obtain the desired structured wave front. In the second stage, the frequency of the incident 803-nm beam is converted to a visible wavelength using SFG in a periodically poled Magnesium-doped lithium niobate (Mg:ppLN) crystal. Finally, the spatial characteristics of the sum-frequency mode are analyzed via far-field intensity measurements, and a coherent mode projection technique using a single-mode fiber. Each stage is discussed in more detail in the following.

### Encoding the phase structure

The input mode was generated from a fiber-coupled 803-nm continuous-wave laser diode. The light emanating from the single-mode fiber was collimated its polarization state was set using a fiber polarization controller. The horizontally polarized 803-nm Gaussian beam was reflected off a spatial light modulator (SLM) and the first-order diffraction was modulated in phase and amplitude^55^ in order to generate a desired spatial mode. Specifically, we generated either spiral phase 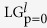 modes^51^ with a radial mode index p =0, superpositions of 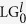 and 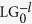, or 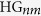 modes, where *n* and *m* are the mode indices. A spatial filter removed the zero-th order diffraction from the SLM before directing the spatially modulated 803-nm beam to the frequency conversion stage. For each of the aforementioned input modes, we verified the expected far-field intensity profiles by inserting a CCD located in the Fourier plane of a 2-f imaging system (not depicted in Fig. 1).

### Sum frequency generation

The frequency converter is based on collinear type-0 SFG in a 10-mm-long Mg:ppLN crystal (Covesion Ltd.) with a poling period of 7.8 μm. The interacting fields are all polarized vertically (parallel to the crystal’s optical z-axis) and propagate along the x-axis in non-critical quasi-phase matching configuration. This avoids the introduction of wave-front distortions due to spatial walk-off, and it allows access to the largest nonlinear coefficient of LN (*d*_33_), which leads to overall higher conversion efficiency. The crystal was maintained at a temperature of approximately 85°C, for SFG with quasi-phase matched center wavelengths of 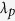 = 1540 nm, 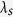 = 803 nm, and 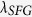 = 527 nm.

A fiber-coupled 1540-nm continuous-wave laser diode was amplified to a power of approximately 60 mW via an Erbium-doped fiber amplifier (measured at the output of the single-mode fiber). A fiber polarization controller and a polarizing beam splitter cube were used to control the power of the pump beam. The pump beam was magnified and combined with the input beam via a dichroic mirror which was highly reflective at 803 nm and highly non-reflective at 1540 nm. The required vertical polarization states of the pump and input mode were set using zero-order half-wave plates (HWP). For the mixing process to occur in the Fourier plane, both beams were focused to the center of the nonlinear crystal using an achromatic lens with a focal length *f * = 100 mm. The beam waists of the Gaussian pump beam was 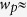 56 μm and the waist parameter of the signal modes was 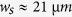, respectively. The SFG was collected using an *f*  = 200-mm lens, and the pump light was guided to a beam dump using a dichroic mirror. The remaining pump light and the 803-nm signal were then blocked using a short-pass filter and a narrowband interference filter with a passband of approximately 10 nm, centered around 532 nm.

### Detection

A flip mirror directed the 527-nm sum-frequency beam to a module for either a phase- or an intensity measurement. The intensity measurements were performed in the far field using a CCD located at the Fourier plane of a 2-f imaging system. The phase structure of the outgoing beam was analyzed by projecting onto an appropriately tailored spatial mode^56^,^57^,^58^. The overlap with the 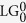 mode, which has a flat phase front, was evaluated by monitoring the power coupled into a single-mode fiber. For the projection onto a 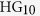 mode, an additional phase mask was inserted before the single-mode fiber. The phase mask was a transparent microscope slide, which was inserted half-way into the beam path. The microscope slide was slightly tilted to obtain the required phase flip of π. The 803-nm input mode was characterized separately, using an additional phase mask and a single-mode fiber (not depicted in Fig. 1).

### Frequency conversion of OAM superposition states

**Figure 2:**
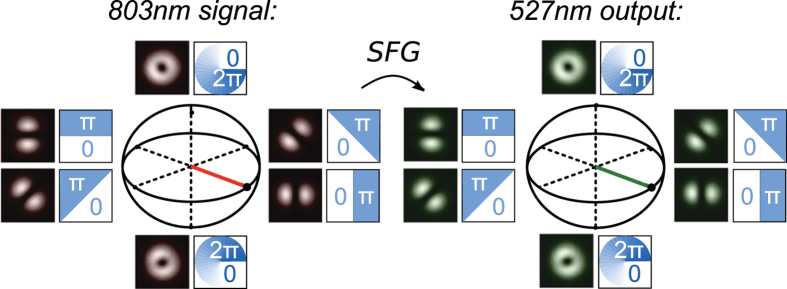
Experimental results visualized on the Poincaré sphere for OAM. The far-field intensity profiles of the 803-nm input beam (left, red) and the frequency-converted 527-nm output beam (right, green) are depicted for several phase profiles applied via the SLM in the 803-nm input beam (inset). The relative phase of the 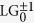 modes determines the angular orientation of the 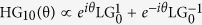 modes on the equator of the Poincaré sphere for OAM.

Our results can be visualized using a Poincaré sphere equivalent for OAM^59^,^60^ (Fig. 2). The polar points on the sphere correspond to 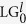 modes with 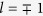. The points on the equatorial plane correspond to OAM superposition states 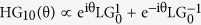. These superpositions correspond to a 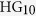 mode, which is rotated by an angle θ 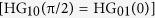. By changing the relative phase and magnitude of the OAM components one can access states distributed over the entire Poincaré sphere. For all phase profiles applied to the input beam, the 527-nm frequency-converted SFG field exhibits the same characteristic far-field intensity profile as the 803-nm input mode (inset Fig. 2). Similar results were obtained for measurements in the image plane (not depicted in Fig. 2.). The fact that the angular orientation of the sum-frequency mode is the same as that of the signal beam demonstrates that the relative phase of the OAM states is conserved in the frequency-conversion process.

For 60 mW of pump power (47 mW impinging on the nonlinear crystal), we observed power-conversion efficiencies (*η*_*p*_ = P_SHG_/P_signal_) of 0.11% for the 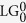 input mode (not depicted in Fig. 2), 0.09% for the 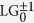 modes, and 0.08% for the 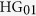 and 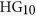 modes. The quantum conversion efficiencies 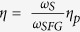 were 0.07% for the 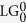 input mode, 0.06% for the 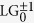 modes, and 0.05% for 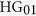 and 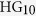 modes, respectively. These results were obtained for a moderate pump power, and the setup can be expected to achieve quantum efficiencies >1% for a pump power of 1 W. At 1550 nm, pump powers >1 W are readily obtained using commercially-available Erbium-doped fiber amplifiers. Achieving higher conversion efficiencies is the subject of further research, and we believe that significant improvements can be expected by placing the nonlinear crystal inside an optical cavity as was done by Zhou *et al*.^49^, or by optimizing the alignment and confocal parameters of the pump- and signal beams.

**Figure 3:**
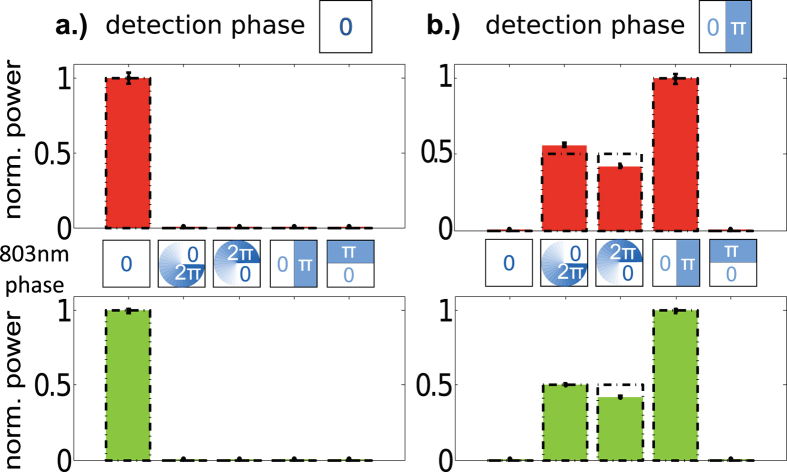
Demonstration of the transfer of the phase structure to the SFG output beam. The bar plots depict the normalized power measured after mode projection with a uniform-phase mask (**a**) or a phase-flip mask (**b**) for various input modes (red, top) and SFG output modes (green, bottom). The error bars due to power fluctuations are small in comparison to the systematic errors due to imperfect alignment of the SLM and projection mode. The dashed line (black) depicts the expected theoretical behavior.

In this proof-of-concept experiment, however, our primary goal was to demonstrate that phase coherence was maintained in the frequency-conversion process. While this claim is already supported by the results discussed in the preceding paragraph, we also performed phase-dependent measurements using a coherent mode projection technique. As a first step, we evaluated the overlap of both the input beam and the SFG beam with the approximately Gaussian mode of a single-mode fiber. Figure 3(a) depicts the normalized fiber-coupled power for various phase profiles applied to the input beam via the SLM. As expected, only the flat phase front of the 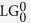
input beam led to efficient coupling into the respective single-mode fiber. Conversely, 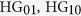, and 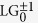 modes showed negligible overlap with the 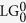 projection mode.

Similar behavior was observed when a phase mask with a π phase step was added before the projection fiber. This approximately corresponds to the projection onto the anti-symmetric 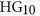 mode (Fig. 3(b)). With the phase mask in place, the 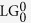 mode resulted in a negligible amount of power coupling into the single mode fiber. In this case, maximal single-mode fiber-coupling efficiency was observed for a 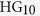 mode, for which the phase mask effectively flattens the wave front. The 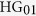 mode, on the other hand, showed negligible overlap with the projection mode. This behavior is expected due to the orthogonality of the HG modes. Since the 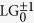 modes are equal superpositions of 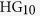 and 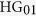 modes we expect the single-mode fiber-coupling to decrease by 50% with respect to the 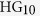 mode. The experimental fiber-coupling efficiencies were slightly unbalanced for 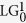 and 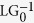, due to misalignment of the SLM and the projection fiber (see Fig. 3(b)).

In summary, the same characteristic behavior was observed for both the input and SFG modes. From these results we can conclude that the phase relationship between different OAM modes is conserved, which demonstrates the suitability of the scheme for coherently converting OAM superposition states for applications in quantum information processing.

### Frequency conversion of higher-order spatial modes

**Figure 4:**
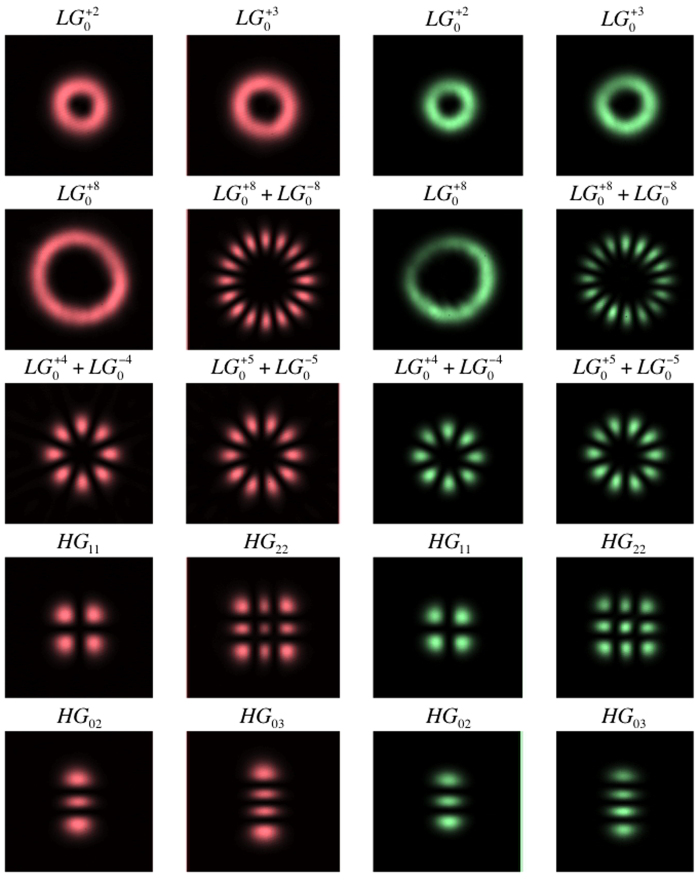
Measured far field intensity profiles of various higher-order spatial modes for the signal beam (left) and the frequency-converted beam (right). The fidelity of the frequency-converted spatial intensity patterns demonstrates the feasibility of coherently frequency converting complex coherent spatial patterns, as well as higher-dimensional quantum systems.

In order to extend these results to higher OAM values and further assess the feasibility of applying the technique, for example, in the coherent imaging of more complex phase structures, we also evaluated the frequency-converted intensity profiles for several higher-order spatial modes. The spatial intensity profiles (Fig. 4) of the 527-nm mode exhibited a high degree of similarity with the 803-nm input mode. However, for higher mode numbers aberrations became apparent. This is the result of crystal inhomogeneity, beam clipping effects at the limited aperture of the nonlinear crystal, as well as spatial filtering effects due to the beam waist of the pump laser. This effect could, however, be mitigated using both a larger crystal aperture, as well as a larger beam waist. Determining the pump focus parameters which lead to the highest conversion-efficiency for a particular higher-order spatial mode, as well as the transfer function for more general distributions of transverse momenta should be the subject of further research.

## Discussion

We have demonstrated coherent frequency conversion of structured light, specifically Laguerre-Gauss and Hermite-Gauss modes, from the near infrared to the visible via the process of sum-frequency mixing in a periodically poled Mg:LN crystal. Our results demonstrate conclusively that the relative phase between Laguerre-Gauss modes with different OAM content is maintained in the frequency-conversion process. This establishes the feasibility of frequency converting quantum information encoded in OAM superposition states for applications in higher-dimensional quantum information processing protocols.

More generally, this can be seen as a method for trans-frequency imaging of a phase-only object, and we believe that more complex phase-structured optical beams may be converted using similar techniques. This could be of great relevance to novel applications in microscopy, since it allows acquiring phase information about an object from light at a wavelength different from that used to illuminate the object^61^.

However, further improvements are necessary in order to obtain higher conversion efficiencies as required for wide-scale practical applicability in different scenarios. Nevertheless, our results demonstrate the experimental feasibility of proof-of-concept experiments involving the coherent frequency conversion of multi-dimensional quantum information encoded in the spatial degree of freedom of (entangled) single photons, which could be of great significance for future applications in quantum information processing and sensing.

The approach used in this article could also be applied to optical parametric amplification, thus enabling the generation of desired spatial modes with high power from a weak seed field. Furthermore, with rapid advancements in nonlinear materials science, it is also foreseeable that similar techniques may be used to mediate parametric photon-photon interactions for complete Bell-state analysis in high-dimensional spatial teleportation protocols and entanglement swapping^62^,^63^.

## Additional Information

**How to cite this article**: Steinlechner, F. *et al*. Frequency conversion of structured light. *Sci. Rep.* **6**, 21390; doi: 10.1038/srep21390 (2016).

## References

[b1] Torres J. P. & Torner L. Twisted Photons: Applications of Light with Orbital Angular Momentum. (John Wiley & Sons, 2011).

[b2] Yao A. M. & Padgett M. J. Orbital angular momentum: origins, behavior and applications. Adv. Opt. Photonics 3, 161–204 (2011).

[b3] Allen L., Padgett M. J. & Babiker M. The orbital angular momentum of light. Prog. Opt. 291–372 (1999).

[b4] Wang J. . Terabit free-space data transmission employing orbital angular momentum multiplexing. Nat. Photonics 6, 488–496 (2012).

[b5] Grier D. G. A revolution in optical manipulation. Nature 424, 810–816 (2003). 12917694 10.1038/nature01935

[b6] Hell S. W. Far-Field Optical Nanoscopy. Science 316, 1153–1158 (2007). 17525330 10.1126/science.1137395

[b7] Fickler R. . Quantum Entanglement of High Angular Momenta. Science 338, 640–643 (2012). 23118185 10.1126/science.1227193

[b8] Barreiro J. T., Langford N. K., Peters N. A. & Kwiat P. G. Generation of Hyperentangled Photon Pairs. Phys. Rev. Lett. 95, 260501 (2005). 16486324 10.1103/PhysRevLett.95.260501

[b9] Hendrych M. . Experimental estimation of the dimension of classical and quantum systems. Nat. Phys. 8, 588–591 (2012).

[b10] Malik M. . Direct measurement of a 27-dimensional orbital-angular-momentum state vector. Nat. Commun. 5, 3115 (2014). 24445503 10.1038/ncomms4115

[b11] Wang X.-L. . Quantum teleportation of multiple degrees of freedom of a single photon. Nature 518, 516–519 (2015). 25719668 10.1038/nature14246

[b12] Kumar P. Quantum frequency conversion. Opt. Lett. 15, 1476–1478 (1990). 19771127 10.1364/ol.15.001476

[b13] Vandevender A. P. & Kwiat P. G. High efficiency single photon detection via frequency up-conversion. J. Mod. Opt. 51, 1433–1445 (2004).

[b14] Ma L., Slattery O. & Tang X. Single photon frequency up-conversion and its applications. Phys. Rep. 521, 69–94 (2012).

[b15] Briegel H.-J., Dür W., Cirac J. I. & Zoller P. Quantum Repeaters: The Role of Imperfect Local Operations in Quantum Communication. Phys. Rev. Lett. 81, 5932–5935 (1998).

[b16] Lvovsky A. I., Sanders B. C. & Tittel W. Optical quantum memory. Nat. Photonics 3, 706–714 (2009).

[b17] Sangouard N., Simon C., de Riedmatten H. & Gisin N. Quantum repeaters based on atomic ensembles and linear optics. Rev. Mod. Phys. 83, 33–80 (2011).

[b18] Ding D. S., Zhou Z. Y., Shi B. S. & Guo G. C. Nat. Commun. 4, 2527 (2013). 24084711 10.1038/ncomms3527 PMC3806433

[b19] Nicolas A., Veissier L., Giner L., Giacobino E., Maxein D. & Laurat J. Nat. Photon. 8, 231–238 (2014). 10.1364/OL.38.000712 23455274

[b20] Ding D. S., Zhang W., Zhou Z. Y., Shi S., Xiang G. Y., Wang X. S., Jiang Y. K., Shi B. S. & Guo G. C. Phys. Rev. Lett. 114, 050502 (2015). 25699427 10.1103/PhysRevLett.114.050502

[b21] Parigi V., D’Ambrosio V., Arnold C., Marrucci L., Sciarrino F. & Laurat J. Nat. Commun. 6, 7706 (2015). 26166257 10.1038/ncomms8706 PMC4510965

[b22] Albota M. A., Wong F. N. C. & Shapiro J. H. Polarization-independent frequency conversion for quantum optical communication. J. Opt. Soc. Am. B 23, 918–924 (2006).

[b23] Ramelow S., Fedrizzi A., Poppe A., Langford N. K. & Zeilinger A. Polarization-entanglement-conserving frequency conversion of photons. Phys. Rev. A 85, 013845 (2012).

[b24] VanDevender A. P. & Kwiat P. G. Quantum transduction via frequency upconversion (Invited). J. Opt. Soc. Am. B 24, 295–299 (2007).

[b25] Boyd R. W. Nonlinear optics. (Acad. Press, 2003).

[b26] Dam J. S., Pedersen C. & Tidemand-Lichtenberg P. High-resolution two-dimensional image upconversion of incoherent light. Opt. Lett. 35, 3796–3798 (2010). 21082000 10.1364/OL.35.003796

[b27] Dam J. S., Tidemand-Lichtenberg P. & Pedersen C. Room-temperature mid-infrared single-photon spectral imaging. Nat. Photonics 6, 788–793 (2012).

[b28] Pedersen C., Karamehmedovic E., Dam J. S. & Tidemand-Lichtenberg P. Enhanced 2D-image upconversion using solid-state lasers. Opt. Express 17, 20885–20890 (2009). 19997325 10.1364/OE.17.020885

[b29] Li X. . Frequency up-conversion imaging with 60-dB gain using picosecond optical parametric amplifier. Chin. Opt. Lett. 11, 111901 (2013).

[b30] Ding D.-S. . Experimental up-conversion of images. Phys. Rev. A 86, 033803 (2012).

[b31] Gurski T. R., Epps H. W. & Maran S. P. Astronomical demonstration of an infrared upconverter. Nature 249, 638–639 (1974).

[b32] Abbas M. M., Kostiuk T. & Ogilvie K. W. Infrared upconversion for astronomical applications. Appl. Opt. 15, 961–970 (1976). 20165103 10.1364/AO.15.000961

[b33] Midwinter J. E. Image conversion from 1.6 μm to the visible in lithium niobate. Appl. Phys. Lett. 12, 68–70 (1968).

[b34] Devaux F. & Lantz E. Parametric amplification of a polychromatic image. J. Opt. Soc. Am. B 12, 2245–2252 (1995).

[b35] Devaux F. . Picosecond parametric amplification of a monochromatic image. Nonlinear Opt 11, 25–37 (1995).

[b36] Devaux F. & Lantz E. Transfer function of spatial frequencies in parametric image amplification: experimental analysis and application to picosecond spatial filtering. Opt. Commun. 114, 295–300 (1995).

[b37] Devaux F. & Passier R. Phase sensitive parametric amplification of optical vortex beams. Eur. Phys. J. D 42, 133–137 (2007).

[b38] Choi S.-K., Vasilyev M. & Kumar P. Noiseless Optical Amplification of Images. Phys. Rev. Lett. 83, 1938–1941 (1999).

[b39] Kolobov M. I. & Lugiato L. A. Noiseless amplification of optical images. Phys. Rev. A 52, 4930–4940 (1995). 9912835 10.1103/physreva.52.4930

[b40] Vaughan P. M. & Trebino R. Optical-parametric-amplification imaging of complex objects. Opt. Express 19, 8920–8929 (2011). 21643145 10.1364/OE.19.008920

[b41] Dholakia K., Simpson N. B., Padgett M. J. & Allen L. Second-harmonic generation and the orbital angular momentum of light. Phys. Rev. A 54, R3742–R3745 (1996). 9914027 10.1103/physreva.54.r3742

[b42] Courtial J., Dholakia K., Allen L. & Padgett M. J. Second-harmonic generation and the conservation of orbital angular momentum with high-order Laguerre-Gaussian modes. Phys. Rev. A 56, 4193–4196 (1997).

[b43] Zhou Z.-Y. . Highly efficient second harmonic generation of a light carrying orbital angular momentum in an external cavity. Opt. Express 22, 23673–23678 (2014). 25321833 10.1364/OE.22.023673

[b44] Zhou Z.-Y. . Generation of light with controllable spatial patterns via the sum frequency in quasi-phase matching crystals. Sci. Rep. 4, 5650 (2014). 25007780 10.1038/srep05650 PMC4090625

[b45] Li Y., Zhou Z.-Y., Ding D.-S. & Shi B.-S. Sum frequency generation with two orbital angular momentum carrying laser beams. J. Opt. Soc. Am. B 32, 407 (2015).

[b46] Ding D.-S., Zhou Z.-Y., Shi B.-S., Zou X.-B. & Guo G.-C. Linear up-conversion of orbital angular momentum. Opt. Lett. 37, 3270–3272 (2012). 22859155 10.1364/OL.37.003270

[b47] Allen L., Beijersbergen M. W., Spreeuw R. J. C. & Woerdman J. P. Orbital angular momentum of light and the transformation of Laguerre-Gaussian laser modes. Phys. Rev. A 45, 8185–8189 (1992). 9906912 10.1103/physreva.45.8185

[b48] Zhou Z.-Y. . Orbital angular momentum light frequency conversion and interference with quasi-phase matching crystals. Opt. Express 22, 20298–20310 (2014). 25321240 10.1364/OE.22.020298

[b49] Zhou Z.-Y. . Orbital angular momentum photonic quantum interface. ArXiv:14107543 Phys. Physics:quant-Ph (2014). at http://arxiv.org/abs/1410.7543 10.1038/lsa.2016.19 PMC6059842 30167117

[b50] Torres J. P., Banaszek K. & Walmsley I. A. in Progress in Optics (ed. Wolf E.) 56, 227–331 (Elsevier, 2011).

[b51] Torres J. P., Alexandrescu A. & Torner L. Quantum spiral bandwidth of entangled two-photon states. Phys. Rev. A 68, 050301 (2003).

[b52] Dam J. S., Pedersen C. & Tidemand-Lichtenberg P. Theory for upconversion of incoherent images. Opt. Express 20, 1475–1482 (2012). 22274491 10.1364/OE.20.001475

[b53] Shao G., Wu Z., Chen J., Xu F. & Lu Y. Nonlinear frequency conversion of fields with orbital angular momentum using quasi-phase-matching. Phys. Rev. A 88, 063827 (2013).

[b54] Delaubert V., Lassen M., Pulford D. R. N., Bachor H.-A. & Harb C. C. Spatial mode discrimination using second harmonic generation. Opt. Express 15, 5815 (2007). 19532840 10.1364/oe.15.005815

[b55] Bolduc E., Bent N., Santamato E., Karimi E. & Boyd R. W. Exact solution to simultaneous intensity and phase encryption with a single phase-only hologram. Opt. Lett. 38, 3546–3549 (2013). 24104810 10.1364/OL.38.003546

[b56] Mair A., Vaziri A., Weihs G. & Zeilinger A. Entanglement of the orbital angular momentum states of photons. Nature 412, 313–316 (2001). 11460157 10.1038/35085529

[b57] Qassim H. . Limitations to the determination of a Laguerre–Gauss spectrum via projective, phase-flattening measurement. J. Opt. Soc. Am. B 31, A20–A23 (2014).

[b58] Hermosa N., Rosales-Guzmán C., Pereira S. F. & Torres J. P. Nanostep height measurement via spatial mode projection. Opt. Lett. 39, 299–302 (2014). 24562131 10.1364/OL.39.000299

[b59] Padgett M. J. & Courtial J. Poincaré-sphere equivalent for light beams containing orbital angular momentum. Opt. Lett. 24, 430 (1999). 18071529 10.1364/ol.24.000430

[b60] Jack B. . Entanglement of arbitrary superpositions of modes within two-dimensional orbital angular momentum state spaces. Phys. Rev. A 81, 043844 (2010).

[b61] Lemos Barretto G. . Quantum imaging with undetected photons. Nature 512, 409–412 (2014). 25164751 10.1038/nature13586

[b62] Kim Y.-H., Kulik S. P. & Shih Y. Quantum Teleportation of a Polarization State with a Complete Bell State Measurement. Phys. Rev. Lett. 86, 1370–1373 (2001). 11178086 10.1103/PhysRevLett.86.1370

[b63] Ether D. S., Walborn S. P. & Zagury N. Complete teleportation of a paraxial single-photon field. Phys. Rev. A 79, 032305 (2009).

